# Drastic Synergy of Lovastatin and *Antrodia camphorata* Extract Combination against PC3 Androgen-Refractory Prostate Cancer Cells, Accompanied by AXL and Stemness Molecules Inhibition

**DOI:** 10.3390/nu15214493

**Published:** 2023-10-24

**Authors:** Chih-Jung Yao, Chia-Lun Chang, Ming-Hung Hu, Chien-Huang Liao, Gi-Ming Lai, Tzeon-Jye Chiou, Hsien-Ling Ho, Hui-Ching Kuo, Ya-Yu Yang, Jacqueline Whang-Peng, Shuang-En Chuang

**Affiliations:** 1 Department of Medical Education and Research, Wan Fang Hospital, Taipei Medical University, Taipei 11696, Taiwan; yao0928@tmu.edu.tw; 2 Department of Internal Medicine, School of Medicine, College of Medicine, Taipei Medical University, Taipei 11031, Taiwan; 101255@w.tmu.edu.tw; 3 Division of Hematology and Medical Oncology, Department of Internal Medicine, Wan Fang Hospital, Taipei Medical University, Taipei 11696, Taiwan; lancehu7@gmail.com (M.-H.H.); gminlai@canceraway.org.tw (G.-M.L.); 108178@w.tmu.edu.tw (T.-J.C.); jqwpeng@nhri.org.tw (J.W.-P.); 4 Cancer Center, Wan Fang Hospital, Taipei Medical University, Taipei 11696, Taiwan; a2639264@ms25.hinet.net (C.-H.L.); injection63@gmail.com (H.-L.H.); ghost1132@yahoo.com (H.-C.K.); 5 National Institute of Cancer Research, National Health Research Institutes, Miaoli 35053, Taiwan; irelandfish@nhri.edu.tw

**Keywords:** lovastatin, *Antrodia camphorata*, prostate cancer, PC3, AXL

## Abstract

Prostate cancer (PC) is the second most frequently diagnosed cancer and the fifth leading cause of cancer-related death in males worldwide. Early-stage PC patients can benefit from surgical, radiation, and hormonal therapies; however, once the tumor transitions to an androgen-refractory state, the efficacy of treatments diminishes considerably. Recently, the exploration of natural products, particularly dietary phytochemicals, has intensified in response to addressing this prevailing medical challenge. In this study, we uncovered a synergistic effect from combinatorial treatment with lovastatin (an active component in red yeast rice) and *Antrodia camphorata* (AC, a folk mushroom) extract against PC3 human androgen-refractory PC cells. This combinatorial modality resulted in cell cycle arrest at the G0/G1 phase and induced apoptosis, accompanied by a marked reduction in molecules responsible for cellular proliferation (p-Rb/Rb, Cyclin A, Cyclin D1, and CDK1), aggressiveness (AXL, p-AKT, and survivin), and stemness (SIRT1, Notch1, and c-Myc). In contrast, treatment with either AC or lovastatin alone only exerted limited impacts on the cell cycle, apoptosis, and the aforementioned signaling molecules. Notably, significant reductions in canonical PC stemness markers (CD44 and CD133) were observed in lovastatin/AC-treated PC3 cells. Furthermore, lovastatin and AC have been individually examined for their anti-PC properties. Our findings elucidate a pioneering discovery in the synergistic combinatorial efficacy of AC and clinically viable concentrations of lovastatin on PC3 PC cells, offering novel insights into improving the therapeutic effects of dietary natural products for future strategic design of therapeutics against androgen-refractory prostate cancer.

## 1. Introduction

Prostate cancer (PC) has been the second-most commonly diagnosed cancer and the fifth leading cause of cancer death in males worldwide [1]. Although androgen deprivation therapy remains the mainstay first-line treatment for PC patients, most patients eventually become “androgen refractory” (resistant to androgen-ablation therapy) within a few years after the initial response [2,3]. Due to the scarcity of efficacious therapeutic options, addressing androgen-refractory PC remains a formidable clinical challenge [4]. Despite significant advances in currently approved treatment options, only a marginal extension of patient survival has been achieved [3,5]. More effective treatment strategies to combat this lethal disease are urgently needed.

Statins, the 3-hydroxy-3-methyl-glutaryl-CoA (HMG-CoA) reductase inhibitors such as FDA-approved simvastatin, atorvastatin, pravastatin, and lovastatin, are the most commonly prescribed drugs for cardiovascular diseases due to their abilities to disrupt cholesterol synthesis and to mitigate arterial cholesterol plaque accumulation [6,7,8,9]. Recently, increasing studies have been initiated to investigate the anti-cancer properties of statins, either alone or in combination with radiotherapy or chemotherapy [10,11,12]. A recent meta-analysis study indicates that using statins in cancer patients can significantly lower cancer-related risk and mortality [13]. Another study by John Hopkins Hospital shows that statins may attenuate PC progression and protect patients from relapse after prostatectomy [14]. Among the statins, lovastatin (Mevacor) is a naturally occurring active ingredient (monacolin K) contained in red yeast rice, a Chinese fermented rice product (*Monascus purpureus*) [15]. This small natural compound (MW 404 Da) is a widely renowned medicine for cholesterol reduction with staggering safety profiles [16]. An investigation carried out by Park et al. [17] demonstrates that lovastatin-induced death of PC3 human androgen-refractory prostate cancer cells occurs via abating transcription factor E2F-1 and its downstream signaling molecules such as c-Myc, cyclin D1, cyclin A, and cyclin B1. In line with this study, Hoque et al. [18] also report that lovastatin can induce apoptosis and G1 phase arrest while reducing cellular levels of phospho-Rb (p-Rb), cyclin D1, cyclin D3, CDK4, and CDK6 in PC3 cells. Owing to the constraints of clinically attainable concentrations of lovastatin [19], it is recommended that further research explore the combinatorial effects of statins and other apoptosis-inducing agents to evaluate their potency against prostate cancer [18].

*Antrodia camphorata* (AC), also known as *Antrodia cinnamomea* or by its Chinese nomenclature “Niu-Chang-Chih”, is an edible Taiwanese mushroom that has been traditionally employed in folk medicine to treat various health disorders [20]. Extracts of AC are reported to possess various biological activities, including anti-inflammatory, antioxidant, hepatoprotective, antihypertensive, antihyperlipidemic, immunomodulatory, and anticancer activities [20]. In fact, the anticancer effects of AC extract have been shown to induce apoptosis and arrest the cell cycle at a concentration of around 150 μg/mL in PC cells [21,22]. Although cyclin B1 and CDK1 (CDC2) reductions are observed in 150 μg/mL of AC extract-treated PC cells, only a limited degree of apoptosis can be induced [22]. AC thus holds promise as a potential adjuvant anticancer agent for treating prostate cancers. Nevertheless, whether its therapeutic potency can be augmented with other dietary components still requires further investigation before optimal integration into clinical practice.

In this study, therefore, we systemically examined whether combining AC extract with clinically achievable concentrations of lovastatin could lead to synergistic efficacy against human androgen-refractory PC cells. The compelling therapeutic effects exerted by AC and lovastatin were molecularly dissected by investigating changes in the protein expression of genes liable for cellular proliferation (p-Rb/Rb, Cyclin A, Cyclin D1, and CDK1), aggressiveness (AXL, p-AKT, and survivin), and stemness (SIRT1, Notch1, and c-Myc) (CD44 and CD133) in PC cells.

## 2. Materials and Methods

### 2.1. Cell Culture

Human androgen-refractory prostate cancer cell lines PC3 (bone-metastatic) [23] and DU145 (brain-metastatic) [24] were maintained in RPMI-1640 (Gibco, Carlsbad, CA, USA) and DMEM (Gibco) medium, respectively. HS68 primary human foreskin fibroblast cells were cultured in DMEM (Gibco) medium. The mediums were supplemented with 10% fetal bovine serum (Gibco) and 1 × penicillin streptomycin-glutamine (Gibco). All cells were cultured at 37 °C in a water-jacketed, 5% CO_2_ incubator. 

### 2.2. Preparation of Lovastatin and AC Extract

Lovastatin was purchased from Calbiochem (San Diego, CA, USA) and was dissolved in dimethyl sulfoxide (DMSO) to make a 10 mM stock solution. The crude material of *Antrodia camphorata* (AC) was provided by Well Shine Biotechnology Development Co. (Taipei, Taiwan) and was extracted by 95% ethanol in a 1:20 (*w*/*v*) ratio for 24 h at room temperature with shaking. The supernatant of extracts was centrifuged at 3000 rpm for 30 min to remove the precipitate, further filtered by a 0.22 micrometer pore size filter (Merck Millipore, Carrigtwohill, Cork, Ireland), and was then lyophilized and stored at −20 °C before use. The final product of the AC extract was reconstituted in 50% dimethyl sulfoxide (DMSO) and 50% EtOH to make a 100 mg/mL stock solution. The stock solutions of lovastatin and AC extract were diluted with culture medium before being added to cultured cells.

### 2.3. Sulforhodamine B (SRB) Assay for Cell Viability

PC3 and DU145 prostate cancer cells were seeded in a 96-well plate at a density of 2 × 10^3^ cells/well. The primary human foreskin fibroblast HS68 cells were seeded in a 96-well plate at a density of 8 × 10^3^ cells/well. After 24 h of incubation at 37 °C, cells were treated with various agents as indicated in the figure legends for a further 72 h. Cells were then harvested and fixed in 10% trichloroacetic acid (TCA). The fixed cells were washed with distilled water and stained with 0.4% (*w*/*v*) SRB dye dissolved in 1% acetic acid. The unbound dye was washed away by 1% acetic acid, and the plates were then air-dried. The cell-bound SRB dye was dissolved by adding 200 μL per well of 10 mM Tris-base, and the absorbance was measured at 570 nm. The absorbance is directly proportional to the cell number over a wide range.

### 2.4. Photograph of the Cells

The images of cells were photographed using a digital microscope camera PAXcam2+ (Midwest Information Systems, Inc., Villa Park, IL, USA) adapted to an inverted microscope CKX31 (Olympus Co., Tokyo, Japan).

### 2.5. Cell Cycle Analysis

A total of 2 × 10^5^ PC3 cells per well was plated on 6-well plates and incubated for 24 h, and then treated with lovastatin (2 μM) and AC extract (40 μg/mL) individually or in combination for 24, 48, and 72 h. At harvest, cells were trypsinized using Trypsin-EDTA (Invitrogen, Carlsbad, CA, USA), washed twice with ice-cold phosphate-buffered saline (PBS), and fixed in cold 70% ethanol overnight at 4 °C. The cells were washed twice with PBS and incubated with 100 μg/mL of propidium iodide (PI) containing 100 μg/mL of RNase at 37 °C for 30 min, and then stored on ice protected from light. Cell cycle analysis was performed by flow cytometry (Beckman Coulter EPICS XL, Fullerton, CA, USA). To evaluate the changes more accurately in distribution at G0/G1, S, and G2/M phases, the percentage of cell-cycle distribution was re-calculated after excluding the sub-G1 proportion.

### 2.6. Western Blotting 

PC3 cells were seeded in 10 cm dishes at a density of 4 × 10^5^ cells/dish for 24 h and then treated with agents as described in the figure legends. On the day of harvest, total protein extracts (10 μg each) were size-fractionated electrophoretically by a 10% polyacrylamide SDS-PAGE gel and transferred onto a PVDF membrane using the BioRad Mini Protean electrotransfer system. The blots were incubated with 5% milk in PBST (phosphate buffered saline with 0.05% Tween-20) for 1 h to block nonspecific binding and incubated with individual primary antibodies overnight at 4 °C, respectively. The primary antibodies purchased from Cell Signaling Technology, Inc. (Danvers, MA, USA) include phospho-Rb (p-Rb) (#9308), SIRT1 (#8469), p-AKT (#9271), and survivin (#2808); antibodies obtained from Abcam, Inc. (Cambridge, MA, USA) include GAPDH (ab8245), CDK4 (ab68266), CD44 (ab51037), and Notch1 (ab52627); those purchased from Santa Cruz Biotechnology (San Diego, CA, USA) include Rb (sc-102), Cyclin A (sc239), CDK-2 (sc-6248), Cyclin D1 (SC-246), AXL (sc-1096), and c-Myc (sc-40); while CDK1 (CDC2) (#1161-1) is obtained from Epitomics (Burlingame, CA, USA); CD133 (#18470-1-AP) is obtained from Proteintech (Chicago, IL, USA). The membranes were incubated with an appropriate peroxidase-conjugated secondary antibody at room temperature for 1 h and washed intensively with PBS. The immune complexes (protein bands) were visualized using an enhanced chemiluminescence detection system (ECL, Perkin Elmer, Waltham, MA, USA) according to the manufacturer’s instructions. The intensities of Western blot bands were quantified using ImageJ software (ImageJ Version 1.52, National Institutes of Health, Bethesda, MD, USA) downloaded from https://imagej.nih.gov/ij/download.html (accessed on 25 September 2019).

## 3. Results

### 3.1. Lovastatin and AC Extract Synergistically Suppress Proliferation and Induce Apoptosis in PC3 Cells

**Figure 1:**
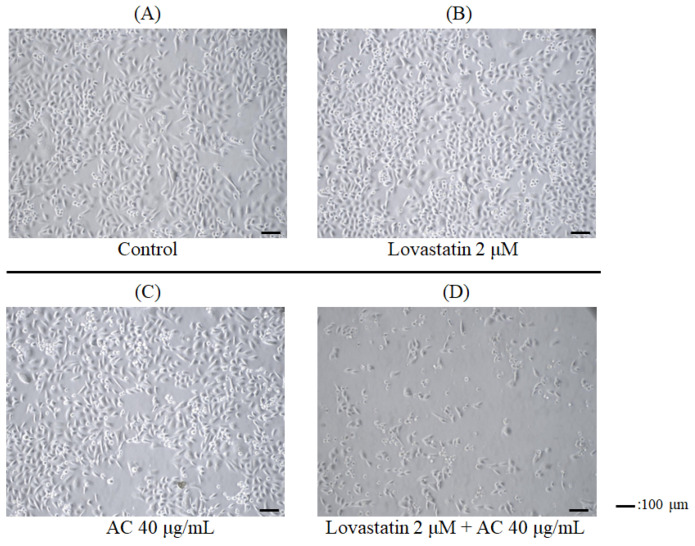
The androgen-refractory prostate cancer PC3 cells were treated with lovastatin and AC extract, individually or in combination, for 72 h. PC3 cells were treated with (**A**) vehicle as the control, (**B**) 2 μM lovastatin, (**C**) 40 μg/mL AC extract, and (**D**) 2 μM lovastatin plus 40 μg/mL AC extract for 72 h. Bar = 100 μm.

**Figure 2:**
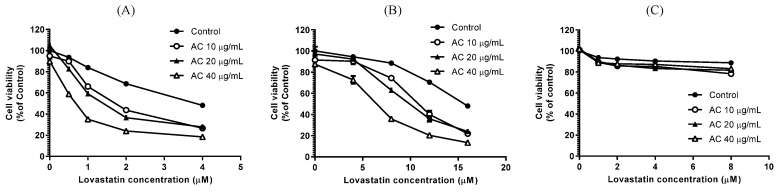
Combination effects of lovastatin and AC extract on cell viability. (**A**) PC3, (**B**) DU145 androgen-refractory prostate cancer cells, and (**C**) HS68 primary human foreskin fibroblast cells were treated as indicated (lovastatin and AC extract, individually or in combination) for 72 h, and then the cell viability was determined by SRB assay as described in Materials and methods.

Firstly, a bone-metastatic androgen-refractory human prostate cancer cell line, PC3, was employed to assess the combinatorial effects of lovastatin and AC extract on cellular proliferation. As shown in Figure 1, lovastatin (2 μM) alone (Figure 1B) and AC extract (40 μg/mL) alone (Figure 1C) showed only marginal impacts on the growth of PC3 cells as compared to the untreated control (Figure 1A). Intriguingly, the combination treatment of both agents dramatically reduced the growth of PC3 cells (Figure 1D). The sulforhodamine B colorimetric (SRB) assay was performed to further demonstrate the synergistic anticancer effect of this combination. After 72 h of treatment, AC extract alone exerted only a minimal inhibitory effect at concentrations of 10 to 40 μg/mL, as cell viability largely remained above 90% (Figure 2A). By contrast, lovastatin alone dose-dependently reduced PC3 cell viability to 48.2% of control at a concentration of 4 μM (Figure 2A). Consistent with this observation, the survival inhibition curve of lovastatin was markedly shifted downward in an AC dose-dependent manner (Figure 2A). A similar phenomenon was also observed in another androgen-refractory and bone-metastatic human prostate cancer cell line, DU145 (Figure 2B). Although higher concentrations of lovastatin alone (8 μM and above) were required to suppress the cell viability of DU145 cells compared to PC3 cells, a similar downward shift of the survival inhibition curve was observed when treated in combination with AC extract (Figure 2B). Furthermore, lovastatin and AC extract did not exhibit inhibitory or synergistic effects on the viability of HS68 primary human foreskin fibroblast cells (Figure 2C), suggesting the proliferation-inhibitor effects of the combinatorial treatment modality using lovastatin and AC extract were exclusive to the growth of malignant cells but not normal cells. 

**Figure 3:**
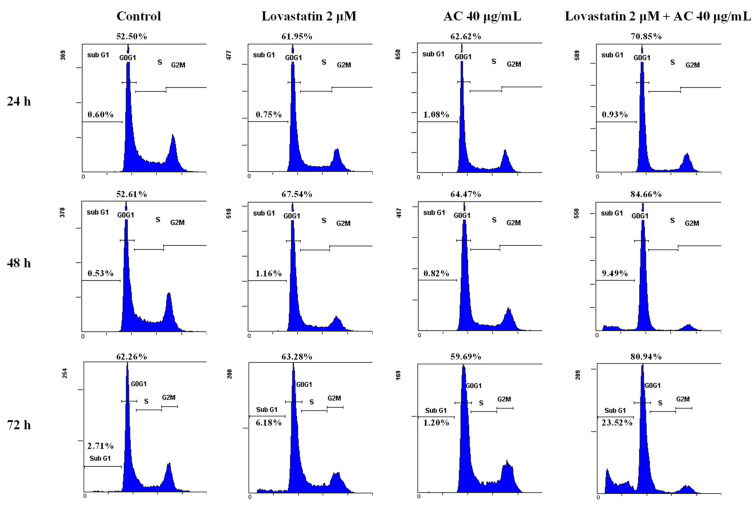
Combination effects of lovastatin and AC extract on the distribution of cell-cycle phase and induction of apoptotic sub-G1 fraction in PC3 cells. Cells were treated as indicated for 24, 48, and 72 h. The percentages of the G0/G1 and sub-G1 fractions are shown in the respective flow cytometric histograms.

The combined effect of lovastatin and AC extract was further investigated by assessing alterations in the cell cycle of PC3 cells. As shown in Figure 3, apoptosis induction and G1 phase arrest were found in the combined treatment group after 72 h, while the effects of lovastatin (2 μM) and AC extract (40 μg/mL) alone on apoptosis or G1 arrest were insignificant (Figure 3). The cell cycle distributions of these lovastatin and/or AC-treated cells are shown in Table 1. In accordance with the findings shown in Figure 1 and Figure 2A, the sub-G1 arrest population was significantly augmented when PC3 cells were treated with lovastatin (2 μM) and AC (40 μg/mL) at both 48 and 72 h, indicating potent combinatorial efficacy from lovastatin and AC extract.

### 3.2. Lovastatin and AC Extract Synergistically Reduce the Proteins Crucial for Cell Cycle Progression in PC3 Cells

**Figure 4:**
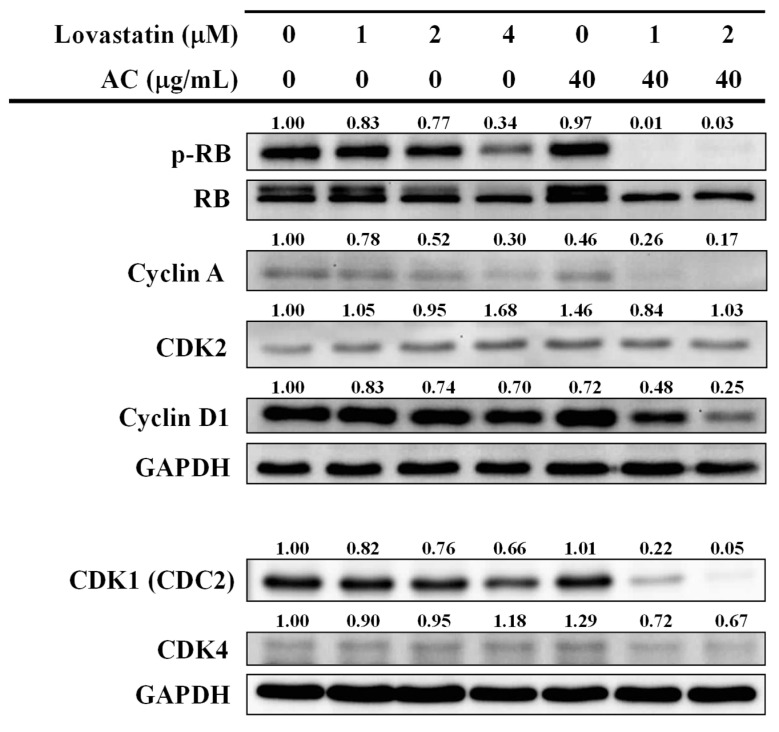
Combination effects of lovastatin and AC extract on cell cycle-regulating molecules in PC3 cells. Cells were treated as indicated for 72 h. Whole-cell lysates were analyzed by Western blot for the proteins of interest. The numbers above the bands indicate the relative densitometric ratios to the bands of loading control (GAPDH). Protein size (kDa): RB (110), Cyclin A (54), CDK2 (33), Cyclin D1 (37), CDK1 (34), CDK4 (34), and GAPDH (35).

To extrapolate the potential mechanism that underlines the novel therapeutic potency exerted by the combination modality of lovastatin and AC through cell cycle regulation in PC3 cells, we next assessed modulations in the expression of proteins crucial for cell cycle progression such as Rb (retinoblastoma protein), p-Rb (phospho-Rb), cyclin A, cyclin D1, CDK1, CDK2, and CDK4. Rb protein is known to play a central role in cell cycle regulation, and its inactivation by phosphorylation, which triggers uncontrolled proliferation, is most common in human sporadic cancers [25]. As expected, the phosphorylation level of Rb was greatly reduced in cells treated with AC extract (40 μg/mL) combined with lovastatin (1 or 2 μM), while individual lovastatin showed only limited (1 or 2 μM) or moderate (4 μM) suppression (Figure 4). Similar changes were also observed in the super-shifted (low-mobility) bands detected in the total Rb blot panel (Figure 4), correlating to the inhibition of Rb phosphorylation. Further, we also revealed marked suppression in cyclin A, cyclin D1, and CDK1 protein levels in a combinatorial group compared to those treated individually (Figure 4). In contrast, protein levels of CDK2 and CDK4 were not markedly affected even in the combined treatment groups, suggesting cyclin A, cyclin D1, and CDK1 as the primary molecules accountable for Rb-mediated cell cycle regulation by the lovastatin/AC combinatorial modality. 

### 3.3. Lovastatin and AC Extract Synergistically Diminish AXL and Survivin in PC3 Cells

**Figure 5:**
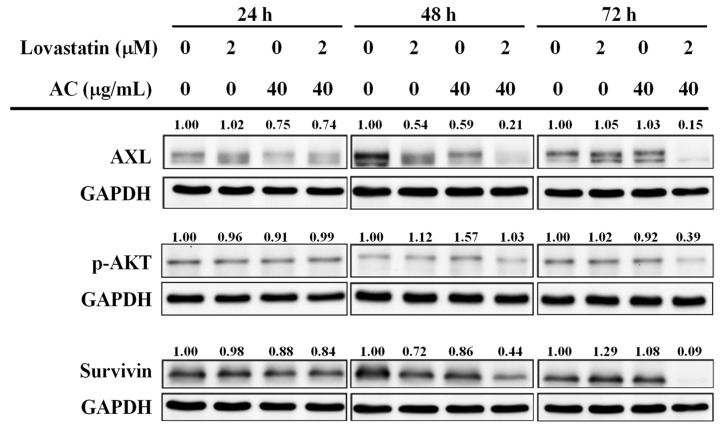
Combination effects of lovastatin and AC extract on aggressiveness-related molecules in PC3 cells. Cells were treated as indicated for 24, 48, and 72 h. Whole-cell lysates were analyzed by Western blot for the proteins of interest. The numbers above the bands indicate the relative densitometric ratios to the bands of loading control (GAPDH). Protein size (kDa): AXL (140), AKT (60), Survivin (16), and GAPDH (35).

In addition to malignant cellular proliferation, aggressive behavior of prostate cancer cells has also been a critical factor contributing to the disease progression [26]. Receptor tyrosine kinase AXL (from the Greek word anexelekto, or uncontrolled) and its downstream phospho-AKT (p-AKT), for instance, are reportedly associated with the aggressiveness and progression of PC [27,28]. Our data demonstrated that expression levels of AXL and p-AKT were dramatically decreased only in PC3 cells treated with a combination of lovastatin and AC extract in a time-dependent manner at 48 and 72 h (Figure 5, upper and middle panels). Apart from AXL, survivin is also frequently associated with biologically aggressive prostate carcinoma [26]. In line with the findings from Figure 4 and AXL, p-ATK levels from Figure 5 and combinatorial lovastatin/AC treatment also predominantly suppressed protein levels of survivin in PC3 cells (Figure 5, lower panel). The aforementioned inhibition of AXL and survivin might profoundly constrain the biologically aggressive behavior of PC3 cells.

### 3.4. Lovastatin and AC Extract Synergistically Suppress Stemness Molecules and Markers in PC3 Cells

**Figure 6:**
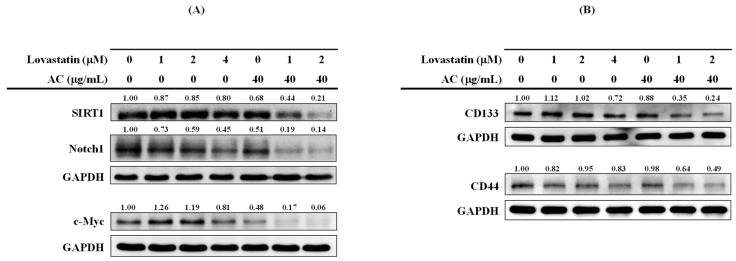
Combination effects of lovastatin and AC extract on the (**A**) stemness molecules and (**B**) stemness markers of PC3 cells. Cells were treated as indicated for 72 h. Whole-cell lysates were analyzed by Western blot for the proteins of interest. The numbers above the bands indicate the relative densitometric ratios to the bands of the loading control (GAPDH). Protein size (kDa): SIRT1 (120), Notch1 (125), c-Myc (67), and GAPDH (35).

Recent research hypothesizes that the primary contributors to distant metastasis and treatment failure in PC are the prostate cancer stem cells, which possess self-renewal properties and elicit resistance to conventional anticancer therapies [29]. Previous studies have shown that SIRT1, Notch1, c-Myc, and downstream targets of miR-34a are positively associated with prostate cancer stem cell traits [30,31]. Next, we analyzed the protein levels of SIRT1, Notch1, and c-Myc in PC3 cells after combinatorial treatment with lovastatin and AC extract to substantiate its potential merits in treating PC. In agreement with their combination effect on the aggressiveness of PC3 cells (Figure 5), lovastatin and AC extract consistently exhibited synergistic suppression in protein levels of SIRT1, Notch1, and c-Myc as compared with individual treatment (Figure 6A). Moreover, in addition to CD44, a direct target of miR-34a [30,31], CD133 is also reported to be an important marker for prostate cancer stem cells [29]. Our data in Figure 6B revealed substantial downregulation of CD133 and CD44 levels in the combinatorial groups but not in the individual treatment groups. Reductions of these stemness molecules and markers might implicate that the synergistically reduced PC proliferation and aggressiveness are attributed to diminished cancer stem subpopulation in lovastatin/AC-treated PC3 cells.

## 4. Discussion

Among the male population in the US, prostate cancer remains the predominant malignancy [30]. Due to the marginal survival benefits that can be offered by current standard-of-care therapies for metastatic androgen-refractory PC patients [30], alternative treatment options like drug repurposing, medicinal plants, and traditional medicine have also been explored in order to fulfill the unmet medical need [5]. The naturally occurring lovastatin from red yeast rice and the edible Taiwanese mushroom *Antrodia camphorata* [15,16,20] have been individually investigated for their effects against prostate cancer, but disappointingly, no clinically viable effects of either compound on prostate cancer have been conclusively reported till now. Results from this study successfully demonstrated synergistic efficacies from a combinatorial strategy using both lovastatin and AC extract, eliciting phenomenal suppression effects on proliferation, aggressiveness, and stemness in PC3 androgen-refractory prostate cancer cells (Figure 1, Figure 2, Figure 5 and Figure 6). Based on the findings presented in our study, lovastatin and AC extract could jointly target molecules responsible for cellular proliferation, aggressiveness, and stemness in PC, providing a plausible explanation for the previous research failure to utilize lovastatin or AC extract alone. This synergism might promote its potential for clinical applications by reducing the required effective concentrations. 

Canonically, the tumor suppressor protein retinoblastoma (RB) crucially regulates cell-cycle progression and proliferation by repressing the E2F1-mediated transcriptional program [32,33,34]. In parallel with our results on the dephosphorylation and thus activation of RB (Figure 4), lovastatin was reported to repress E2F1 and induce apoptosis of PC3 cells at a concentration of 10 μM by Park et al. [17]. In fact, higher concentrations (above 10 μM) required for inducing lovastatin-mediated cytotoxicity were also reported by two previous studies in PC cells [35,36]. Considering that 3.92 μM is the maximum plasma lovastatin concentration detected in clinical trials [37], lovastatin alone seems unlikely to effectively modulate E2F1 in prostate cancer patients. Likewise, the most effective concentrations of statins in other research papers are far from the clinically viable range [38]. Although the anticancer effects of statins against prostate cancer had been intensively studied [39,40], the hurdle remained, thus becoming the choice of a combinatorial compound that could potentiate the therapeutic efficacy of lovastatin to achieve a clinically viable concentration. In our study, an edible compound like AC extract and its renowned safety profile [16] were chosen to investigate its combinatorial anticancer effect with lovastatin against PC.

Multiple oncogenic transcription factors, including but not limited to E2F1, will be rewired after the loss of RB function [34,41]. In addition to regulating the cell cycle, RB plays a pleiotropic role in cancer restriction [34]. Recent studies have declared that RB loss-of-function is tightly associated with aggressive disease and poor outcomes in PC [33,34,41]. In combination with AC extract, lovastatin dephosphorylated and thus activated RB in PC3 cells at concentrations below 3.92 μM. Our Figure 5 showed a reduction of AXL and survivin, two known aggressiveness-regulating molecules of prostate cancer [26,27,28,42], by combinatorial lovastatin and AC extract, which echoed the roles of RB in cancer control. Survivin is originally known as an IAP (inhibitor of apoptosis) protein [26]. The induction of the apoptotic sub-G1 fraction in lovastatin/AC extract-treated PC3 cells might largely be attributed to the profound decrease of survivin in these cells. 

Further, it is noteworthy that AXL is reportedly overexpressed in prostate cancer cell lines and human prostate tumors [42], and its expression is considerably higher in more aggressive androgen-refractory PC3 and DU145 cells compared with the androgen-dependent cell line LNCaP [43]. Consequently, the crucial role of AXL in the progression and metastasis of prostate cancer has made it an attractive therapeutic target [42], as various synthetic inhibitors have been conducted in clinical trials for AXL-targeted therapies [44]. Thus, the drastic AXL-diminishing effect of combining lovastatin with AC extract opened a new avenue to future strategic integration with currently available AXL inhibitors for better treatment against aggressive prostate cancers. 

Furthermore, AXL is also closely associated with epithelial-mesenchymal transition (EMT), drug resistance, and cancer stemness [44]. In lovastatin/AC extract-treated PC3 cells, the greatly reduced AXL levels from Figure 5 thus consistently reflected another observation in ablating stemness molecules (SIRT1, Notch1, and c-Myc) and markers (CD44 and CD133) in Figure 6. Contrary to the expected oncogenic roles of Notch1 and c-Myc, the biological functions of SIRT1 in cancer remain controversial [45]. It is reported that the NAD+-dependent deacetylase, i.e., Sirtuin 1 (SIRT1), possesses both oncogenic and tumor-suppressive functions in PC, possibly depending on the stage of tumor progression in context-dependent manners [46]. Compared with normal prostate epithelial PrEC cells and normal prostate cells obtained from patients, SIRT1 expression is markedly up-regulated in human PC cell lines such as LNCap, PC3, and DU145 cells [47,48]. Regarding the roles of the SIRT1-c-Myc axis in supporting cancer stem cell maintenance [30,31,45,49]. Moreover, the reduction of SIRT1 in lovastatin/AC extract-treated PC3 cells might contribute to not only growth inhibition but also mitigated cancer stemness. Since it has been reported that CD133 is a robust biomarker for prostate cancer stem cells, a combination of CD133+ and CD44+ markers, with or without integrin 21, may further improve the isolation of prostate cancer stem cells from clinical specimens [29]. Statins have been strategically proposed as an anti-cancer stem cell compound. Nonetheless, clinical studies conducted to date have yet to deliver conclusive evidence [50]. Given the aforementioned combination effects of lovastatin and AC extract, the CD133+/CD44+ population might be substantially eliminated in the combinatorial-treated PC3 cells. 

## 5. Conclusions

Despite recent advances, androgen-refractory prostate cancer remains without a definitive cure [3,5]. Our current study exploited a novel therapeutic intervention targeting AXL [44], Notch1 [51], Myc [52], or SIRT1 [46] in an attempt to improve the management of androgen-refractory prostate cancers. The findings from this study underscore the potential of integrating lovastatin with AC extract, providing a synergistic or adjunctive pharmacological strategy to amplify the clinical effectiveness of lovastatin or AC extract alone. Since edible dietary products typically exhibit negligible physiological toxicity, a comprehensive preclinical evaluation is also imperative to optimize the dosage, initiation, sequence, and duration of this novel combinatorial treatment modality warranted by lovastatin and AC extract prior to their clinical use.

## 6. Patents

An invention patent (NO. I 311912, TW) resulted from this work.

## Figures and Tables

**Table 1:** Tabulated cell-cycle distribution analysis (by percentages from Figure 3) of PC3 cells treated with lovastatin and/or AC extract after 72 h.

Lovastatin (μM)	0	2	0	2
AC (μg/mL)	0	0	40	40
Sub-G1 (%)	2.7	6.2	1.2	23.5
G0/G1 (%)	62.2	63.3	59.7	81.0
S (%)	21.7	19.0	18.5	10.4
G2/M (%)	16.1	17.7	21.8	8.6

## Data Availability

Not applicable.
